# Notes and News

**Published:** 1899-04-01

**Authors:** 


### NOTES AND NEWS.

(Collected and Communicated.)

The University of Philadelphia is to have a new pharmacological, physiological, and pathological laboratory, costing 6,000 dollars.

The Right Hon. Sir Charles Hall, K.C.M.G., Recorder of London, will preside at the biennial festival of the National Hospital for the Paralysed at the Hotel Metropole, on May 31st.

A large number of Egyptian pilgrims resort to Mecca, and the sanitary department of the Egyptian Government have taken the precaution to draft regulations to come into force should the plague be introduced into Egypt.

A beautiful statue, representing the finding of Moses, executed by Lombadi, has been presented to the Darlington Hospital by Mrs. J. Backhouse, in memory of the late Mr. and Mrs. Alfred Backhouse. The group has been placed in the entrance hall of the hospital.

At the council meeting on Tuesday of the Medical Graduates' College and Polyclinic an offer by Mr. Jonathan Hutchinson of a loan of £2,000 at small interest was accepted. This loan is to be utilised to erect and equip a museum and laboratory at once on vacant ground adjoining the college.

The German medical students in Berlin are actively opposing the introduction of female medical students into the university and hospitals. They have formulated an appeal which has been placarded on the notice boards of the Berlin University and the clinical hospitals.

Dr. Oliver (of the Home Office Committee on Dangerous Trades), and Professor Thorpe (Principal of the Government Laboratory) have prepared a Blue-book on the results of their investigations upon the poisoning by lead of workers in the Pottery industries. After recording the seriously injurious effect of the use of lead in glazing upon the operators, they recommend that the use of raw lead should be prohibited, and that women, young persons of both sexes, should not be employed in the dipping house where lead is used, nor as ware cleaners after dippers in lead glaze.

A new aspect of the effect of the X rays has shown itself in Paris, where a lady has entered an action against that practice on the part of an operator who, she alleges, scorched her in the course of an investigation.

The site of the proposed colony for epileptics in Cheshire has been purchased at Sandlebridge, near Knutsford. It consists of 450,000 acres, and on it will be erected villas, farm buildings, social hall, and administrative buildings. The David Lewis trustees have granted £50,000 towards this useful scheme, which emanates from Manchester.

With reference to the question of inquiry into the circumstances of applicants for relief, for the purpose of preventing the abuse of charity, we learn that the strong feeling displayed by the working men governors against the system as practised experimentally for the past three months at the City of London Hospital for Diseases of the Chest (Victoria Park), has been met in the following manner. The Committee of Management has decided to accept the stamp, on a subscriber's letter, of any of the recognised working men's associations entitled to representation on the governing body, without further inquiry, thus throwing the responsibility for the fitness of applicants recommended by them upon these societies. The series of questions to which so much exception has been taken is to be deleted from the letters.

Middlesex Hospital Medical School was *en fête* last week (Wednesday, 15th inst.) on the occasion of the inauguration of the new school buildings. Sir Ralph Thomson (chairman of the council), Dr. Cayley (the senior physician), Mr. Andrew Clark (the senior surgeon), and Dr. Pasteur (the Dean) received the guests, who numbered some six or seven hundred, and comprised representatives of the official and medical staffs of most of the metropolitan hospitals. The Middlesex has reason to be proud of its medical school, which, in convenience of arrangement and completeness of equipment, is now second to none. The new buildings will provide ample accommodation for a further 50 students in addition to the 200 attending the existing school; and they will have the advantage of every appliance that science has so far suggested for the furtherance of bacteriological research, chemical investigation, and anatomical and pathological study. Throughout the various rooms were distributed most interesting scientific, surgical, and optical instruments; drawings illustrative of various phases of rare diseases; and microscopical specimens and preparations, as well as practical demonstrations of the various uses to which the more recent adaptations of the Röntgen apparatus can be put. The gathering was a brilliant one, and all concerned are to be congratulated on the success of the function. Music was supplied by the band of the Royal Artillery, and the various exhibits were in charge of members of the staff and others, among whom may be mentioned Professor Ramsay, Dr. Travers, Dr. Voelcker, Mr. Alex. Foulerton, Dr. R. A. Young, Mr. J. S. Goodall, Mr. Somerville Hastings, Dr. Pringle, and Dr. H. Campbell Thomson. Mr. Baldwin, the casualty surgical officer, was especially active in attending to the comfort of guests, and in taking care that they saw all that was best worth seeing.

